# Head & neck acinar cell carcinoma: a population-based study using the seer registry

**DOI:** 10.1186/s12885-020-07066-y

**Published:** 2020-07-08

**Authors:** Feiluore Yibulayin, Lei Feng, Meng Wang, Meng-meng Lu, Yuan Luo, Hui Liu, Zhi-cheng Yang, Alimujiang Wushou

**Affiliations:** 1 Department of Oral & Maxillofacial Surgery and Oral Biomedical Engineering Laboratory Shanghai Stomatological Hospital, Fudan University, 356 Beijing East Road, Shanghai, 200001 People’s Republic of China; 2 Department of Preventive Medicine, School of Public Health, Shanghai Medical College, Fudan University, 138 Yi xue yuan Road, Shanghai, 200001 People’s Republic of China

**Keywords:** Acinar cell carcinoma, Head and neck, Survival, Prognostic factor, SEER database

## Abstract

**Background:**

To explore the clinicopathologic characteristics, treatment and prognostic factors of head and neck acinar cell carcinoma (HNACC) comprehensively.

**Methods:**

A population-based study was conducted using data from the Surveillance, Epidemiology, and End Results database (1975–2016). Overall survival (OS) and HNACC-specific survival of patients with different clinicopathologic variables were compared using the Kaplan-Meier method and Cox multivariate regression.

**Results:**

A total of 2624 primary HNACC cases (1052 males, 1572 females) were identified. There was a significant difference in gender distribution. Among the total cohort, 2416 cases originated from salivary glands, including 2325 parotid gland ACC cases. Regardless of confounding factors, the 10-year and 20-year disease-specific survival (DSS) was 93.6 and 90%, respectively. Surgery was favourably associated with better DSS and OS [HR = 0.13, *P* = 0.0092 and HR = 0.23, *P* = 0.0203]. Gender was the only demographic independent prognostic factor for both DSS and OS [Male vs female, HR = 3.3, *P* = 0.0028 for DSS; HR = 2.44, *P* = 0.0376 for OS]. Higher pathological grade was adversely associated with DSS and OS [Grade II, HR = 4.03, *P* = 0.0444; Grade III + IV, HR = 35.64, *P* = 0.0000 for DSS; Grade III + IV, HR = 4.49, *P* = 0.0000 for OS, Grade I as reference]. In addition, TNM/AJCC stage was commonly associated with prognosis.

**Conclusion:**

Surgery was the only favourable prognostic indicator for both DSS and OS. Gender, age, pathological differentiation and TNM/AJCC stage were independent prognostic factors for survival.

## Background

Salivary gland malignant tumours account for 1–3% of head and neck cancers [1]. Acinar cell carcinoma (ACC) is an uncommon malignant tumour, and its predominant site of origin is salivary glands in the head and neck region [2]. Approximately 80% of ACC originates in the parotid gland; the remaining disease originates in the submandibular and sublingual glands, and there are reports involving the hypopharynx, lip, thyroid, tongue and tonsil [3]. Head and neck ACC (HNACC) is mainly found in the salivary gland, and ACC accounts for only approximately 6% of all salivary gland neoplasms. Apart from mucoepidermoid carcinoma, adenocarcinoma, and adenoid cystic carcinoma, ACC is the fourth most common reported malignancy of the parotid gland. Given the rarity of the disease, data on the general demographics, clinical characteristics, treatment and prognosis of HNACC are still sporadic [4].

There have been no controlled studies that define the optimal treatment for HNACC. The treatment modalities reported including surgical resection, radiotherapy and chemotherapy, and combined therapies with variable results [3]. However, among all salivary gland cancers, HNACC has a relatively favourable prognosis [5]. It is worth noting that, based on some researchers’ institutional experience, HNACC is classified as a low-grade cancer regardless of its clinicopathologic features [6]. Pathological differentiation (high grade vs low grade) is the only considered prognostic indicator [7]. ACC has a tendency to recur locoregionally, to produce lymph node and distant metastasis, and to display aggressive evolution [8].

For rare tumours, nationwide population-based retrospective analysis may make it possible to evaluate trends in demographic features, clinicopathologic characteristics, treatment modalities and disease-specific prognostic indicators. We conducted a comprehensive analysis of all primary HNACC cases registered in the Surveillance, Epidemiology, and End Results (SEER) database of the United States National Cancer Institute from 1975 to 2016.

## Methods

### Study population

SEER*Stat software developed by the National Cancer Institute (Surveillance Research Program, National Cancer Institute SEER*Stat software 8.3.6; https://seer.cancer.gov) was used to extract the study cases. International Classification of Diseases for Oncology (ICD-O-3) codes for acinar cell carcinoma (8550/3) and head and neck topographic codes were used to identify cases with a diagnosis of HNACC registered in the SEER database. The variables in the analysis included marital status at diagnosis, insurance status, tumour orientation, age range, mean age, gender, race, pathological differentiation, American Joint Committee on Cancer (AJCC) stage, TNM stage, neck dissection, surgery, radiotherapy and chemotherapy. Our study used established data and did not involve interaction with human subjects. Therefore, institutional review board approval was not required.

### Statistical analysis

Statistical analysis was performed using the statistical packages R (The R foundation; http://www.r-project.org; version 3.4.3), Empower R (http://www.empowerstats.com, Boston, Massachusetts), and the Statistical Package for the Social Sciences, Version 23.0, for Windows (SPSS, Chicago, IL). Differences in numerical variables were assessed using Student’s t-test or the non-parametric Wilcoxon test. The chi square test or Fisher’s exact test for categorical variables was used for two-group comparisons of parameters. Survival curves for different variable values were generated using Kaplan-Meier estimates and were compared using the log-rank test. Variables that achieved significance at *P* < 0.05 were entered into the multivariable analyses via the Cox regression model.

## Results

### Summary statistics

A total of 2624 primary HNACC cases were included in the study. The demographic and clinicopathological characteristics of the cases are summarized in Table 1. There is a significant difference in gender distribution; 1052 of the cases are male, and 1572 are female. The mean age at diagnosis was 50 years for the total cohort and ranged from 5 to 96 years. According to this dataset, HNACC occurred in the lip, nose and nasal cavity, middle ear, eye and orbit, gum, floor of the mouth, tonsil, tongue, thyroid gland, oropharynx, nasopharynx, hypopharynx, larynx, pharynx, submandibular gland, sublingual gland and parotid gland. Among the total cohort, 2416 cases originated from salivary glands, including 2325 parotid gland ACC cases. White people accounted for 81% of the total population (2131/2624). With respect to pathological differentiation, definite pathological information was available for only 938 cases. Most of the cases were grade I and grade II; grade III plus IV carcinoma accounted for 12.3%. The percentages of cases with lymph node metastasis and distant metastasis were 7% (96/1351) and 6% (76/1183), respectively. Surgical resection was the primary treatment modality. Surgery was performed in 2516 patients, including 905 patients in which simultaneous neck dissection was performed, and 839 cases received surgery plus radiotherapy.

**Table 1:** The summary of HNACC patients’ clinicopathologic characteristics

**Variables**		**Disease specific survival**				**Overall survival**			
		**Alive**	**Dead**	**Total**	***P*-value**	**Alive**	**Dead**	**Total**	***P*-value**
**Marital status at diagnosis**	Single	568	28	596	0.000	569	69	638	0.000
	Married	1086	129	1215		1094	355	1449	
	Other status	204	56	260		204	175	379	
**Insurance status**	Any Medicaid	94	8	102	0.224	94	12	106	0.837
	Insured	866	44	910		873	78	951	
	Uninsured	28	3	31		28	5	33	
**Tumor orientation**	Salivary gland	1845	210	2055	0.175	1854	562	2416	0.002
	Beyond salivary gland	138	10	148		140	68	208	
**Age age**	0–19	171	0	171	0.000	171	3	174	0.000
	20–29	243	5	248		243	10	253	
	30–39	323	15	338		324	34	358	
	40–49	398	32	430		399	66	465	
	50–59	396	41	437		398	103	501	
	60–69	288	58	346		291	152	443	
	70–79	119	36	155		122	163	285	
	80+	45	33	78		46	99	145	
**Gender**	Female	1210	112	1322	0.004	1219	353	1572	0.000
	Male	773	108	881		775	630	1405	
**Race**	Black	193	16	209	0.003	193	45	238	0.000
	White	1570	196	1766		1579	552	2131	
	Others	179	7	186		181	30	211	
**Pathological grade**	Grade I	407	18	425	0.000	409	71	480	0.000
	Grade II	283	20	303		284	59	343	
	Grade III + IV	42	56	98		42	73	115	
**AJCC stage**	Stage I	531	8	539	0.000	532	35	567	0.000
	Stage II	350	12	362		353	28	381	
	Stage III + IV	173	61	234		174	90	264	
**AJCC T stage**	T1	656	17	673	0.000	660	56	716	0.000
	T2	371	20	391		374	41	415	
	T3 + T4	147	48	195		148	72	220	
**AJCC N stage**	N0	1066	49	1115	0.000	1074	113	1187	0.000
	N1	32	18	50		32	24	56	
	N2 + N3	16	17	33		16	24	40	
	NX	60	1	61		60	8	68	
**AJCC M stage**	M0	1111	72	1183	0.000	1119	147	1266	0.000
	M1 + M2	63	13	76		63	22	85	
**Neck dissection**	No	507	22	529	0.020	510	72	582	0.608
	Yes	796	62	858		801	104	905	
**Radiotherapy**	No	1359	98	1457	0.000	1366	389	1755	0.002
	Yes	624	122	746		628	241	869	
**Chemotherapy**	No	1966	203	2169	0.000	1977	610	2587	0.000
	Yes	17	17	34		17	20	37	
**Treatment**	Surgery	1354	96	1450	0.000	1361	386	1747	0.010
	Surgery+RT	611	107	718		615	224	839	

#### Survival analysis

**Fig. 1:**
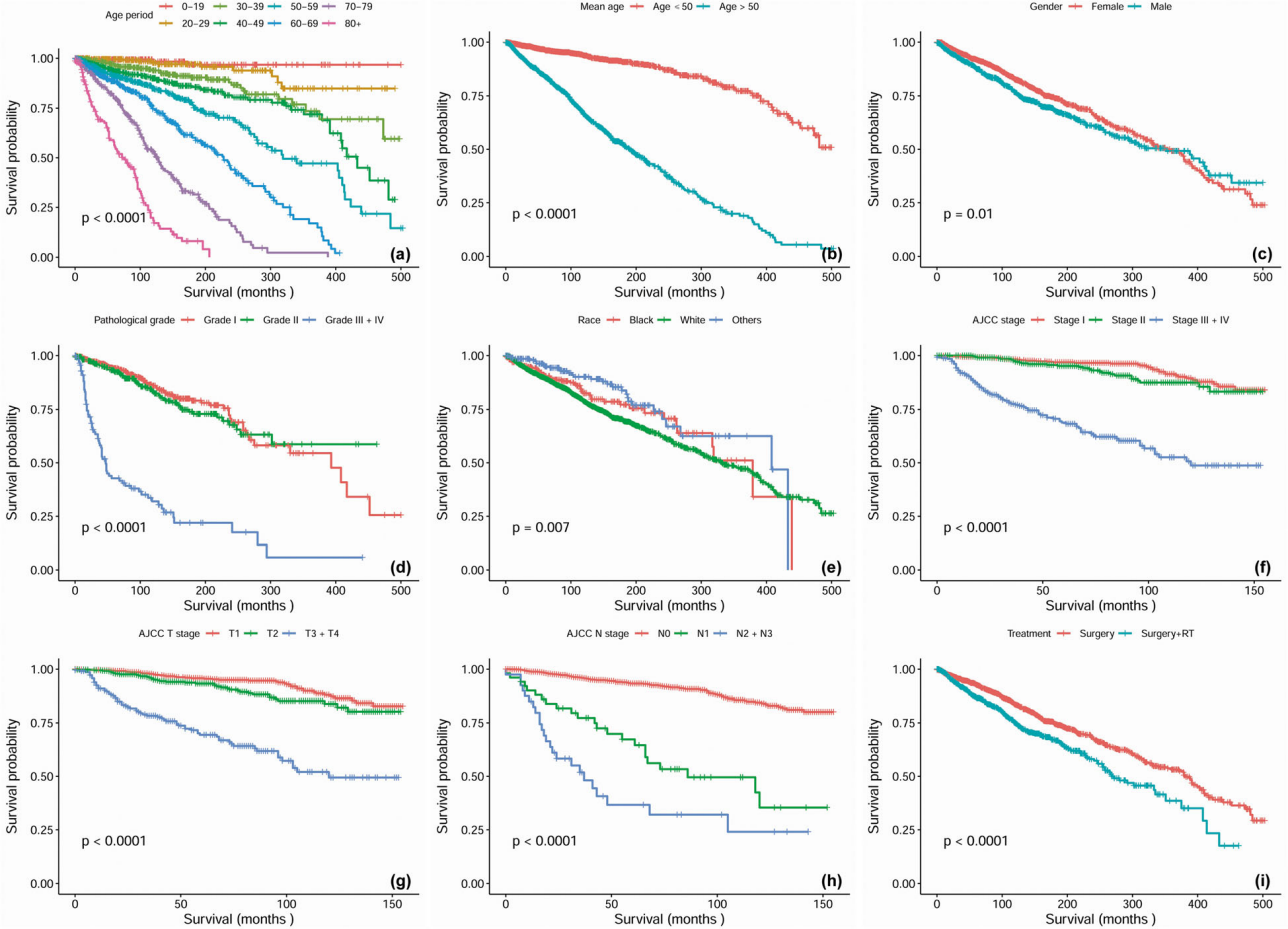
Overall survival curves of cases with HNACC compared according to (**a**) age range, (**b**) mean age, (**c**) gender, (**d**) pathological grade, (**e**) race, (**f**) AJCC stage, (**g**) AJCC T stage, (**h**) AJCC N stage and (**i**) treatment modalities. Log-rank test was utilized to compare curves, and significance is presented on each panel

**Fig. 2:**
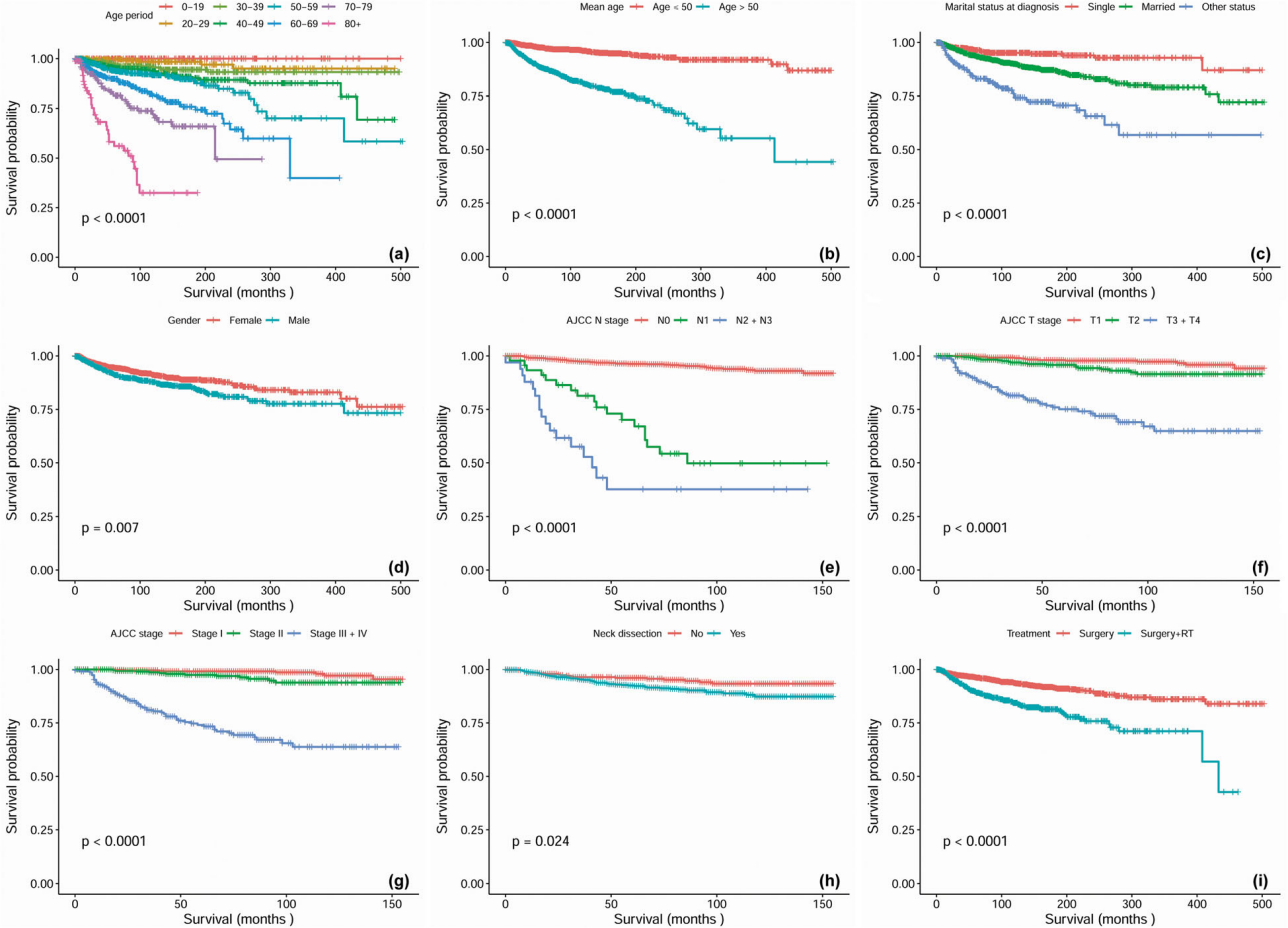
Disease specific survival curves of cases with HNACC compared according to (**a**) age range, (**b**) mean age, (**c**) material status, (**d**) gender, (**e**) AJCC N stage, (**f**) AJCC T stage, (**g**) AJCC stage, (**h**) neck dissection, and (**i**) treatment modalities. Log-rank test was utilized to compare curves, and significance is presented on each panel

Kaplan-Meier analysis was performed for time-to-event analysis for overall survival (OS) and disease-specific survival (DSS). Regardless of confounding factors, 5-year, 10-year and 20-year OS was 90, 80 and 64%, respectively. Significant differences in OS were found depending on age range (*P* < 0.0001), mean age (*P* < 0.0001), marital status at diagnosis (*P* < 0.0001), gender (*P* = 0.01), pathological differentiation (*P* < 0.0001), race (*P* = 0.007), AJCC stage (*P* < 0.0001), AJCC T stage (*P* < 0.0001), AJCC N stage (*P* < 0.0001), AJCC M stage (*P* < 0.0001), surgery, (*P* < 0.0001), radiotherapy (*P* < 0.0001), chemotherapy (*P* < 0.0001) and combined treatment modality (*P* < 0.0001) (Fig. 1). Of the total cohort, 2203 patients were available for DSS analysis. The median follow-up time for these cases was 123 months (range, 1–503 months). Regardless of all other factors, 10-year and 20-year DSS was 93.6 and 90%, respectively, for patients who received surgery alone and 84.3 and 75.8%, respectively, for patients who received surgery plus radiotherapy (*P* < 0.0001). In addition, statistically significant differences in DSS were found to be associated with age (*P* < 0.0001), race (*P* = 0.007), mean age (*P* < 0.0001), marital status at diagnosis (*P* < 0.0001), gender (*P* = 0.007), AJCC stage (*P* < 0.0001), AJCC T stage (*P* < 0.0001), AJCC N stage (*P* < 0.0001), AJCC M stage (*P* < 0.0001), neck dissection (*P* = 0.024) and pathological differentiation (*P* < 0.0001) (Fig. 2).

**Fig. 3:**
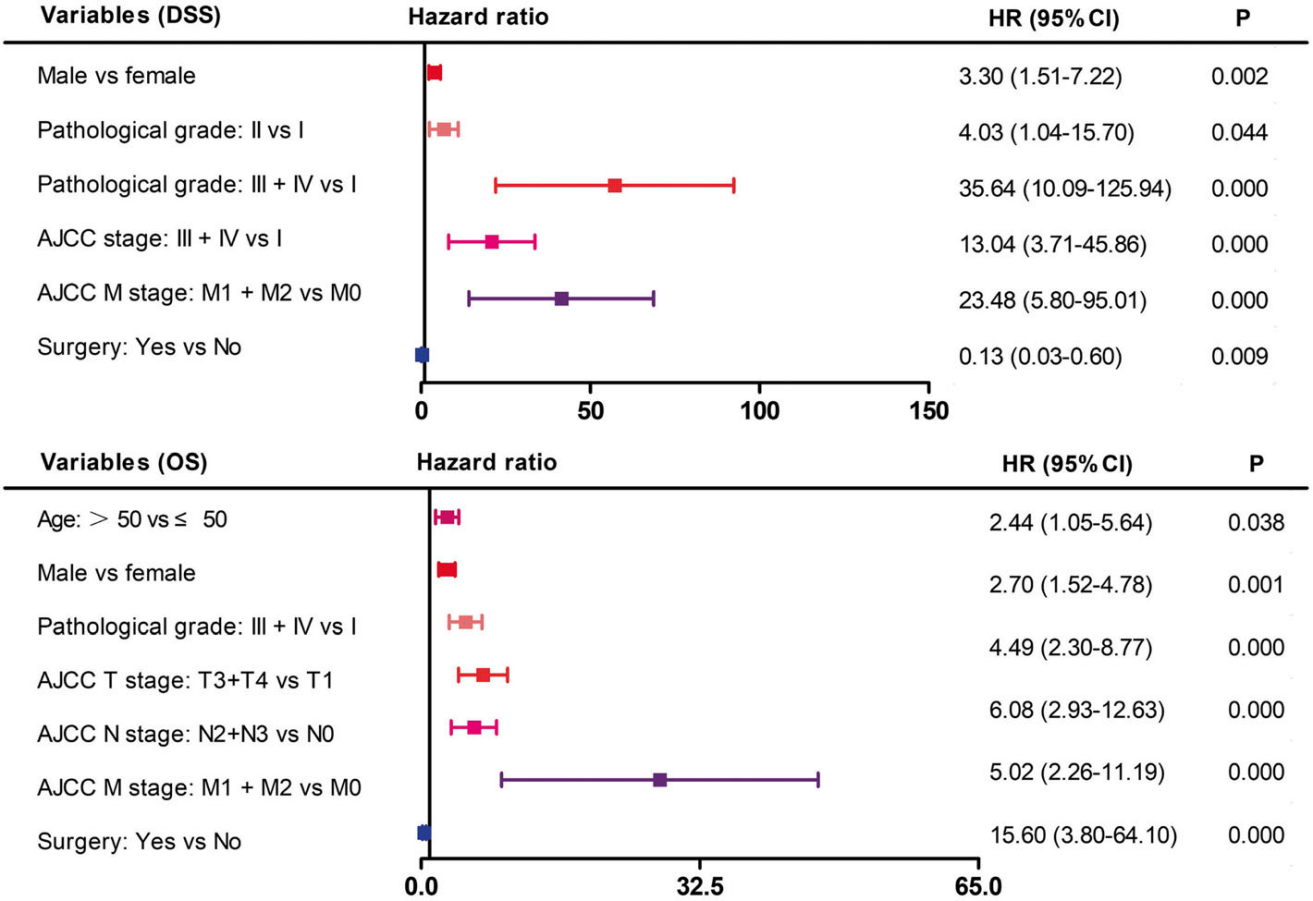
Forest plots summarizing hazard ratios for (**a**) DSS and (**b**) OS

Multivariate survival analysis was performed using the Cox proportional hazards regression model and the significant variables listed above. Surgical treatment was favourably associated with better DSS and OS [HR (95% CI) = 0.13 (0.03–0.6), *P* = 0.0092; HR (95% CI) = 0.23 (0.07–0.79), *P* = 0.0203]. Gender was the only demographic independent prognostic factor for both DSS and OS [Male vs female, HR (95% CI) = 3.3 (1.51–7.22), *P* = 0.0028 for DSS; HR (95% CI) = 2.44 (1.05–5.64), *P* = 0.0376 for OS]. Higher pathological grade was adversely associated with DSS and OS [Grade II HR (95% CI) = 4.03 (1.04–15.7), *P* = 0.0444; Grade III + IV, HR (95% CI) = 35.64 (10.9–125.94), *P* = 0.0000 for DSS; Grade III + IV, HR (95% CI) = 4.49 (2.3–8.77), *P* = 0.0000 for OS, Grade I as reference]. Details of the multivariate Cox regression analysis are presented in Fig. 3.

## Discussion

With the exception of case reports and small retrospective case series, no adequate data describing HNACC demographics are available [4, 9, 10]. In the current investigation, the gender incidence distribution showed a higher number of females than males, and there was a statistically significant predominance of HNACC in females (*P* < 0.001). HNACC could be found at any age, and the mean age at diagnosis was 50 years. Previous research on HNACC is based on single institutional experiences, and because of the small sample size, those studies are often not sufficiently powerful to find considerable differences in survival that are related to general demographic parameters [11–13]. In the survival analysis in our study, significant differences in DSS and OS were found to be related to age range, mean age, gender, race and marital status (*P* < 0.001). The most important findings regarding the demographics of HNACC are that gender and mean age are independent prognostic indicators for DSS and OS and that females under 50 years of age had more favourable prognosis than males over fifty.

TNM/AJCC staging plays an essential role in the planning of tumour treatment and in prognostic evaluation. The lymph node and distant metastasis rates of HNACC are low; the lymph node metastasis rate is less than 10%, and the distant metastasis rate is approximately 5 % [14]. In this circumstance, AJCC T stage plays a crucial role in prognosis evaluation; the larger the volume of the tumour is, the worse the prognosis is. In our results, AJCC T3 + T4 stage is an adverse independent OS prognostic indicator. There is a question of whether elective neck dissection should be performed in HNACC patients. In our DDS analysis, HNACC patients did not benefit from elective neck dissection. Patients with and without elective neck dissection showed no statistically significant differences in OS or in the survival analysis of the pathological grade III + IV subgroup and the AJCC T3 + T4 stage subgroup. In view of the low frequency of metastases, routine elective treatment of the neck is not recommended. However, elective neck dissection should be considered in the treatment of large and high-grade tumours [15].

In previous articles, pathological grade was used as an important prognostic reference [6, 16, 17]. Consistent with earlier reports, our results indicate that pathological differentiation is the strongest prognostic indicator. Compared to well-differentiated Grade I HNACC cases, Grades II, III and IV are unfavourably associated with DSS and OS. The pathologic subtypes of ACC have been reported [3, 18]. Unfortunately, the effects of these variants on prognosis could not be established because of the limited data. HNACC was mainly found in the parotid gland, and surgical resection was the primary treatment modality. Unlike the rest of the head and neck region, because of the presence of the facial nerve, the orientation of parotid gland ACC determines the type of surgery that is performed (Partial parotidectomy, superficial parotidectomy or total parotidectomy). The choice of surgical type has a huge impact on the prognosis of patients with parotid gland malignancies [19, 20]. According to the distribution of sample size and site, we divided the study population into two groups: (a) parotid gland group and (b) beyond parotid gland group. The results of survival analysis show that there was no significant difference in the survival of these two groups. The SEER system does not provide detailed information about the location of parotid gland ACC or the type of surgery performed. Thus, we were unable to perform further analysis based on surgical type. In this cohort, 839 patients received radiotherapy; the prognosis of those patients was poor compared to that of patients who were treated with surgery alone. This result should be interpreted cautiously because in the clinical treatment of parotid gland ACC, radiotherapy is often implemented when adverse factors such as positive surgical margins or poor pathologic differentiation are present [21].

Several important limitations of this study are acknowledged. First, this SEER-based investigation was a retrospective analysis with some inherent bias. Some crucial data such as surgical type, surgical margins, and details of neck dissection could not be obtained, restricting further analysis. A geographic bias could not be totally avoided because of the nonuniform distribution of the SEER registries. Second, retrospective SEER-based analysis depends on accurate coding and consistent data collection among numerous sites, both of which can be imprecise. Third, due to the lack of complete data on adjuvant therapy, the role of radiotherapy and chemotherapy could not be well established. Finally, the retrospective nature of the study and the incompleteness of the clinicopathological data may weaken the strength of our conclusions.

## Conclusion

In summary, despite the limitations of the incomplete data and of the study itself, to the best of our knowledge the present study is the first investigation to use a large study population and a long follow-up time to define the clinicopathologic characteristics and identify the prognostic indicators of ACC in the head and neck region. Three earlier reports described ACC cases reported in the National Cancer Database and the SEER database [2, 5, 14]. Although there is overlap among the study samples, the conclusions are complementary and enhance each other due to the different research intentions. Gender was the only demographic independent prognostic factor for both DSS and OS. With respect to treatment, surgical treatment was the only independent favourable prognostic factor for both DSS and OS. In addition, age, pathological differentiation, and TNM/AJCC stage were associated with DSS and OS.

## Data Availability

Study data was publicly available in the SEER database.

## Abbreviations

HNACC: Head and neck acinar cell carcinoma
SEER: Surveillance, Epidemiology, and End Results
OS: Overall survival
DSS: Disease-specific survival
HR: Hazard ratio
ACC: Acinar cell carcinoma
ICD-O-3: International Classification of Diseases for Oncology
AJCC: American Joint Committee on Cancer
SPSS: Statistical Package for the Social Sciences
